# Evaluating performance of the Bioline™ HCV point-of-care test in Ghana

**DOI:** 10.1186/s12879-025-11730-8

**Published:** 2025-10-15

**Authors:** Evans Duah, Evans Mantiri Mathebula, Kuhlula Maluleke, Daniel Edem Azumah, Richard Kobina Dadzie Ephraim, Tivani Mashamba-Thompson

**Affiliations:** 1 School of Health Systems and Public Health, Faculty of Health Sciences, University of Pretoria, Pretoria, 0002 South Africa; 2 Clinical Laboratory Department, Cape Coast Teaching Hospital, Cape Coast, Ghana; 3 Department of Medical Laboratory Science, College of Health and Allied Sciences, School of Allied Health Sciences, University of Cape Coast, PMB, Cape Coast, Ghana

**Keywords:** Hepatitis C virus, Point-of-care test, Diagnostic performance, Sub-Saharan Africa, Ghana

## Abstract

**Background:**

Hepatitis C Virus (HCV) causes liver diseases including chronic hepatitis, cirrhosis, and hepatocellular carcinoma. In low- and middle-income countries (LMICs), particularly sub-Saharan Africa (SSA), HCV diagnostic resources are limited. Moreover, most evaluations of point-of-care (POC) invitro diagnostics (IVDs) are conducted outside the region using non-African populations, which may not reflect their performance in local settings where they are mostly used. This study assessed the diagnostic performance of the Bioline™ HCV POC test in Ghanaian HCV target populations.

**Methods:**

A cross-sectional field evaluation was conducted among HCV priority populations including incarcerated individuals, patients requiring HCV testing, and voluntary blood donors undergoing pre-donation screening. Venous blood samples were tested using the Bioline™ HCV POC test, and the results were compared with the Enzyme-Linked Immunosorbent Assay (ELISA) reference standard. The sensitivity, specificity, test efficiency, Youden index, predictive values, likelihood ratios, and receiver operating characteristic (ROC) indicators were calculated.

**Results:**

The Bioline™ HCV POC test demonstrated a sensitivity of 96.7% (95% CI: 82.8–99.9%), specificity of 99.8% (95% CI: 98.9–100%), and positive and negative predictive values of 96.7% (95% CI: 82.8–99.9%) and 99.8% (95% CI: 98.9–100%), respectively. The test efficiency was 99.6% (98.6–99.9%), Youden index 0.97 (0.82–0.99) with a ROC area of 0.98 and highly favorable likelihood ratios (LR + 483.5, LR − 0.03).

**Conclusion:**

This study highlights the high diagnostic performance of the Bioline™ HCV POC test in Ghanaian populations. The test’s reliability underscores its potential as a valuable tool for HCV screening and early detection in resource-limited settings, contributing to efforts to reduce the global HCV burden.

**Trial registration:**

This study is part of a diagnostic trial registered in the Pan African Clinical Trial Registry (https://pactr.samrc.ac.za) on 24th October 2024 with trial registration number: PACTR202410837698664.

**Supplementary Information:**

The online version contains supplementary material available at 10.1186/s12879-025-11730-8.

## Introduction

Hepatitis C Virus (HCV) is a major cause of hepatitis and one of the leading reasons for liver transplantation [1, 2]. Globally, more than 58 million out of the over 130 million people with HCV infection progressed to chronic viral hepatitis [2, 3]. The disease disproportionately affects low-and-middle-income countries (LMICs) primarily due to inadequate testing and poor linkage to care [4]. Poor diagnostic resourcing, inadequate external donor support, poor procurement practices and lack of diagnostic quality assurance contribute to inadequate and inaccessible HCV testing algorithms in SSA [5, 6].

The World Health Organization (WHO) recommends mandatory HCV screening, especially for donor blood and blood products intended for transfusion [2], as well as in settings where HCV antibody seroprevalence is ≥ 2% [7]. In addition, screening is recommended for at-risk target-specific populations: migrants from endemic settings, men who have sex with men (MSM), people living with HIV (PLHIV), persons who inject drugs (PWID), healthcare workers, pregnant women and prison inmates [7, 8]. In high income countries, HCV is reliably screened with serological qualitative assays that detect antibodies to HCV (anti-HCV), preferably chemiluminescence immunoassay (CLIA), followed by complementary and confirmatory nucleic acid testing (NAT) [9–11]. However, recommendations to use these testing algorithms may not be feasible and sustainable in under-resourced and hard-to-reach settings in LMICs, especially in SSA. These assays, deployed in high-cost facilities, require laboratory-led technical competencies to operate sophisticated and expensive equipment that operates effectively on a stable electricity supply [12]. The WHO recommends a two-step HCV testing algorithm for priority populations in resource-limited settings and communities inaccessible to laboratory-led central laboratories [13]. This testing strategy requires an initial antibody-based HCV point-of-care (POC) test followed by an HCV NAT-based confirmatory test such as the POC HCV RNA assay [13].

POC rapid in vitro diagnostics serve under-resourced and hard-to-reach communities, especially in LMICs and SSA [14]. However, quality performance issues are commonly raised during the implementation of POC diagnostics [15]. The WHO Prequalification (PQ) process recommends rapid in vitro diagnostics (IVDs) and POC devices to have an accurate performance with a sensitivity of ≥ 98% and a specificity of ≥ 97% [16]. In addition, the WHO PQ process recommends in-country-specific test performance evaluations to establish populations-specific clinical performance to not compromise on quality [16].

Several POC HCV IVDs have been evaluated globally on their diagnostic performance to implement POC, primarily using non-African populations [6]. However, there are limited SSA evaluation records, especially using SSA-specific populations. Most evaluations of the clinical performance of IVDs in SSA are left at the country-specific regulatory agencies’ discretion, which may be limited in their functioning and capacity [17]. The purpose of this study was to evaluate the performance of the Bioline™ HCV POC test in diagnosing HCV among priority population groups as part of a diagnostic trial in Ghana. The Bioline™ HCV test, produced by Abbott Diagnostics, comprises products (02FK10, 02FK16, 02FK17) that are WHO prequalified (PQDx 0257–012-00), carry the CE mark, and are approved for use in Ghana by the Food and Drugs Authority (FDA) under registration number FDA/D.21–11,702 [18, 19]. It is anticipated that the results of this study will benefit healthcare providers with reliable HCV diagnostics, improve HCV early detection for priority groups, and guide policymakers in integrating effective tools into healthcare systems. It also supports global efforts toward HCV elimination, particularly in resource-limited settings.

## Methods

### Study design

This performance evaluation was conducted as the first phase of a more compressive diagnostic evaluation study aimed at evaluating the Bioline™ HCV POC test with guidance from the WHO REASSURED criteria. The protocol of the diagnostic evaluation study is registered in the Pan African Clinical Trial Registry (PACTR202410837698664) whose protocol is published elsewhere [20], The current study evaluated the diagnostic performance of the Bioline™ HCV POC test among Ghanaian populations between August 2024 and December 2024.

### Study setting

**Fig. 1:**
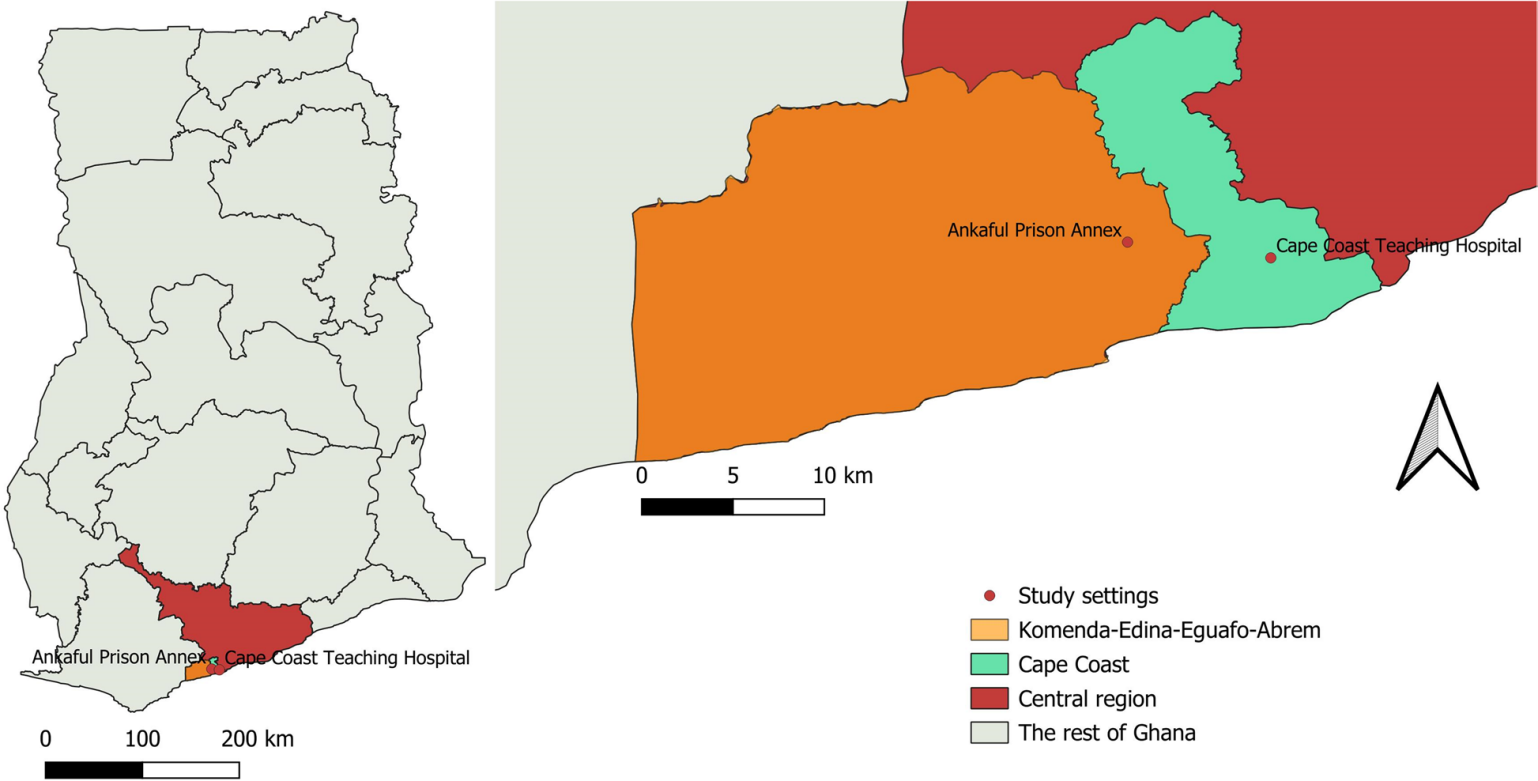
Map of Ghana showing the study settings

Two public institutions located in the Cape Coast Metropolitan and the Komenda-Edina-Eguafo-Abrem (KEEA) districts in Ghana’s Central region were included in the study, thus the Cape Coast Teaching Hospital (5.133134, −1.266336) and the Ankarful Prison Annex (5.14088, −1.33711) respectively (Fig. 1). The decision to include the correctional facility was guided by existing literature emphasizing the risks associated with confined spaces and the potential sharing of sharp objects among incarcerated individuals [8]. On the other hand, the Cape Coast Teaching Hospital is the major referral healthcare facility in the Central region and its environs providing valuable services including blood banking and hepatitis clinic.

### Target population

Three HCV priority populations including incarcerated individuals from the prison, patients with a clinician’s request for HCV test and voluntary blood donors with a request for pre-donation screening from the laboratory department of the Cape Coast Teaching Hospital were included in the study (Table 1). Multiple priority populations were approached to optimize the HCV case detection, given Ghana’s relatively low prevalence (1–3%).

**Table 1:** Characteristics of study participants

Priority population	Facility	Total population size	Sample size	Median age (years) (IQR*)	Sex of participants	
					**Male** **n(%)**	**Female** **n(%)**
Incarcerated individuals	Ankarful Prison Annex	745	219	32(26–38)	219(100.0)	0(0.0)
Patients	Cape Coast Teaching Hospital	500	139	31(21–41)	69(49.6)	70(50.4)
Voluntary blood donors	Cape Coast Teaching Hospital	500	158	31(25–41)	77(48.7)	81(51.3)
Total		1745	516	31(25–40)	365(70.7)	151(29.3)

^*^IQR Inter-quartile range

#### Sample size

The Buderer’s sample size formular for diagnostic evaluations was employed [21]. The minimum total sample size of 433 was required to produce estimates depicting the diagnostic performance of the Bioline™ HCV POC test with a 95% confidence interval and 5% margin of error. Specifically, the field evaluation required a minimum sample size of 399 to estimate the test’s sensitivity using the manufacturer’s pre-determined test sensitivity provided in the test leaflet (Se = 99.3%). Similarly, 34 minimum samples were required to significantly estimate the test’s specificity based on the pre-determined specificity (Sp = 98.1%). The sample size calculations are presented as Supplementary file 1.

### Participant selection strategy

The probability proportional to size (PPS) sampling technique was used to apply proportional stratification, ensuring equal sampling from each target risk group (stratum) based on the size of the stratum. This method ensures that the sample size for each risk group is proportionate to the population size of the stratum. The minimum required sample sizes for each stratum were calculated using the following equation and presented as Supplementary file 2.

$${n}_{i}=\frac{n}{N}x {N}_{i}$$

*n*_*i*_ = Sample size for stratum *i* (the specific HCV risk group)

*N*_*i*_ = Population size of stratum *i*

*n* = Total sample size

*N* = Total population size

The consecutive sampling technique was used to obtain the required sample size for the study. This sampling technique allowed for the inclusion of all eligible participants who met the predefined inclusion criteria and provided consent, ensuring their selection and participation until the required sample size was reached [22].

#### Inclusion criteria

- ▪ At the prison, all incarcerated individuals who had served five years and more and consented to be screened were included in the study. This threshold was set based on the recognition that long-term imprisonment, particularly in facilities with poor conditions, poses significant health and behavioral risks, including increased exposure to infectious diseases and risky practices. Establishing this minimum sentence helped ensure that participants have had sufficient duration of exposure to the prison environment for relevant risk factors to manifest.
- ▪ At the Cape Coast Teaching Hospital, all patients with a clinician’s request for HCV test and voluntary blood donors with a request for pre-donation screening who gave informed consent were included in the study.

#### Exclusion criteria

- ▪ All participants who could not provide adequate venous blood samples were excluded from the study.

### Study procedures

#### Blood sample acquisition and Bioline™ HCV POC testing

In a security-controlled and coordinated environment such as the prison, the principal investigator and trained research assistants, comprising medical laboratory professionals and public health officers, explained the purpose of the study to the participants in both English and Fanti languages. Participants were assured that their participation was entirely voluntary and that refusal to participate would not result in any form of punishment, retaliation, or denial of basic needs, including access to food, healthcare, or other essential services, as such actions would violate their fundamental rights. These assurances were explicitly outlined in the informed consent process. The consent form was read to them in a language they understood, ensuring full comprehension, and each participant either signed or provided a thumbprint to indicate their informed agreement. To protect confidentiality and minimize potential risks, data collection was conducted in a private setting, with only authorized research personnel present. Personal identifiers were anonymized or removed to safeguard participant privacy. Prison officials were not involved in the consent process, preventing any undue influence or coercion. Consenting individuals were permitted to pose additional questions and were answered to their understanding.

**Fig. 2:**
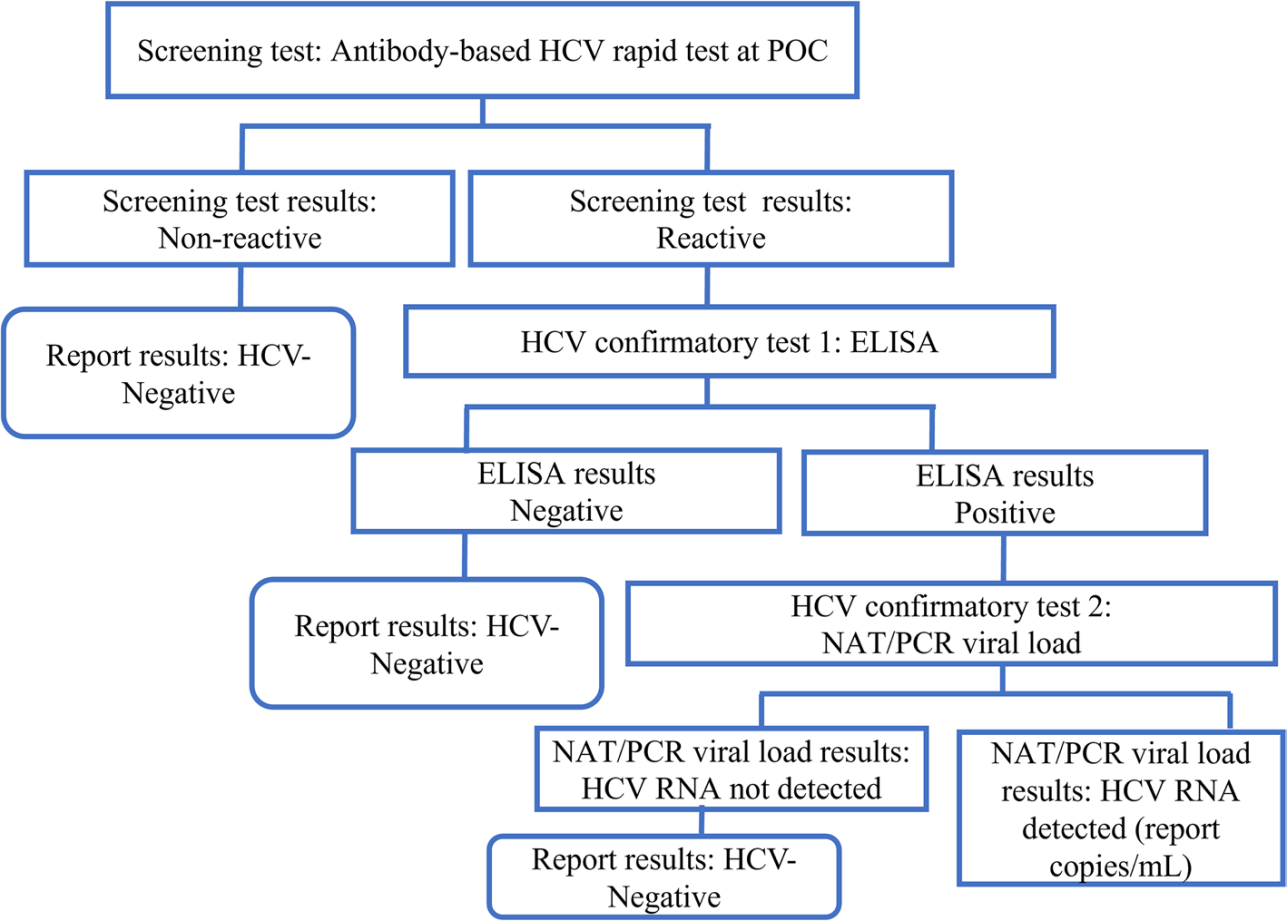
HCV testing algorithm for diagnosing HCV at the Cape Coast Teaching Hospital, Ghana

A researcher-assisted paper-based structured questionnaire was administered to collect non-identifiable socio-demographic information of participants, including age, sex (binary), sexual relationship status, history of sexually transmitted infection, and history of blood transfusion to guide the participant selection based on risk of HCV infection (Supplementary file 3). Additionally, the public health officers conducted physical examinations for tattoos and evidence of injecting drugs. The questionnaire was also read in Fanti for individuals who were unable to comprehend English. The research team provided pre-counseling to the study participants and engaged them in a medical screening under the supervision of the prison’s in-house medical officers. Venous blood samples were collected into a 5 ml gel separator tube and a 4 ml EDTA tube by the medical laboratory professionals. The Bioline™ HCV testing was conducted at POC with the venous blood sample collected in the EDTA tube. Both blood tubes were transported to the Cape Coast Teaching Hospital laboratory and processed according to the study work instructions, standard operating procedures, and recommended testing algorithms (Fig. 2). The serum sample obtained by centrifuging the gel separator tube was used for the reference testing.

Furthermore, at the Cape Coast Teaching Hospital laboratory, whole blood and serum samples were collected from patients who had a clinician’s request for HCV testing. These included patients suspected of having viral hepatitis based on their clinical presentation. In addition, blood samples from voluntary blood donors undergoing pre-donation screening were collected. These samples were collected by medical laboratory professionals. The blood samples, along with non-identifiable demographic information of the patients and donors, were cataloged (Supplementary file 4). Whole blood samples were subsequently analyzed using the Bioline™ HCV POC test.

#### Reference testing

The diagnostic performance of the Bioline™ HCV POC test was evaluated against the Enzyme-linked Immunosorbent Assay (ELISA) available in the Cape Coast Teaching Hospital’s laboratory as the HCV gold standard. The facility uses the ChemWell® Fusion Automatic immunoassay analyzer, which operates with the Fortress Diagnostics qualitative HCV kit. In this study, the CE-marked Fortress Diagnostics qualitative HCV kit (96-test ELISA, Product Code: BXE0781C), manufactured in the United Kingdom, was used [23]. This kit detects serum HCV antibodies (Anti-HCV) using a qualitative chemiluminescence immunoassay on a microplate, based on the sandwich ELISA principle. It demonstrates a sensitivity of 99.79% and a specificity of 99.55%. The test procedure adhered to standard protocols, following the manufacturer’s instructions and the standard operating procedures of the Cape Coast Teaching Hospital laboratory [23, 24]. It involved the use of microwells pre-coated with HCV capture antibodies, blocking of non-specific binding sites, sample incubation, washing, binding with a detection antibody, a second washing step, substrate addition, color development, and result interpretation.

### Referrals for HCV treatment

All ELISA HCV positive cases underwent confirmatory testing with polymerase chain reaction (PCR) and were subsequently linked to treatment at the Cape Coast Teaching Hospital’s hepatitis clinic. However, PCR was not included in the diagnostic performance evaluation.

### Data analysis

Data were entered into Microsoft Excel spreadsheets (Microsoft Office version 2010) and subsequently cleaned to eliminate duplicates, missing data, and errors. The study’s primary outcome was the qualitative HCV test results generated by various diagnostics, indicating the HCV status of the target population included in the study. This outcome was recorded as a binary variable and presented as positive or negative for both Bioline™ HCV POC and ELISA. The cleaned data were exported to the Stata/IC statistical software version 16.0 and labeled for statistical analysis. Performance analysis for diagnostic tests was conducted using the Stata command *“diagt”.* The performance analysis measured the Sensitivity (Se), Specificity (Sp), Positive Predictive Value (PPV), Negative Predictive Value (NPV), Test Efficiency (TE), Youden Index (J), Receiver Operating Characteristic (ROC) area, Positive Likelihood Ratio (LR +), and Negative Likelihood Ratio (LR-) of the Bioline™ HCV POC test with corresponding 95% confidence intervals (CI) [20].

## Results

The study involved 516 participants from three priority populations: incarcerated individuals, patients, and voluntary blood donors. The median age was 31 years (IQR: 25–40). Males accounted for 70.7%, and females made up 29.3%. A summary of the study population is presented in Table 1.

**Fig. 3:**
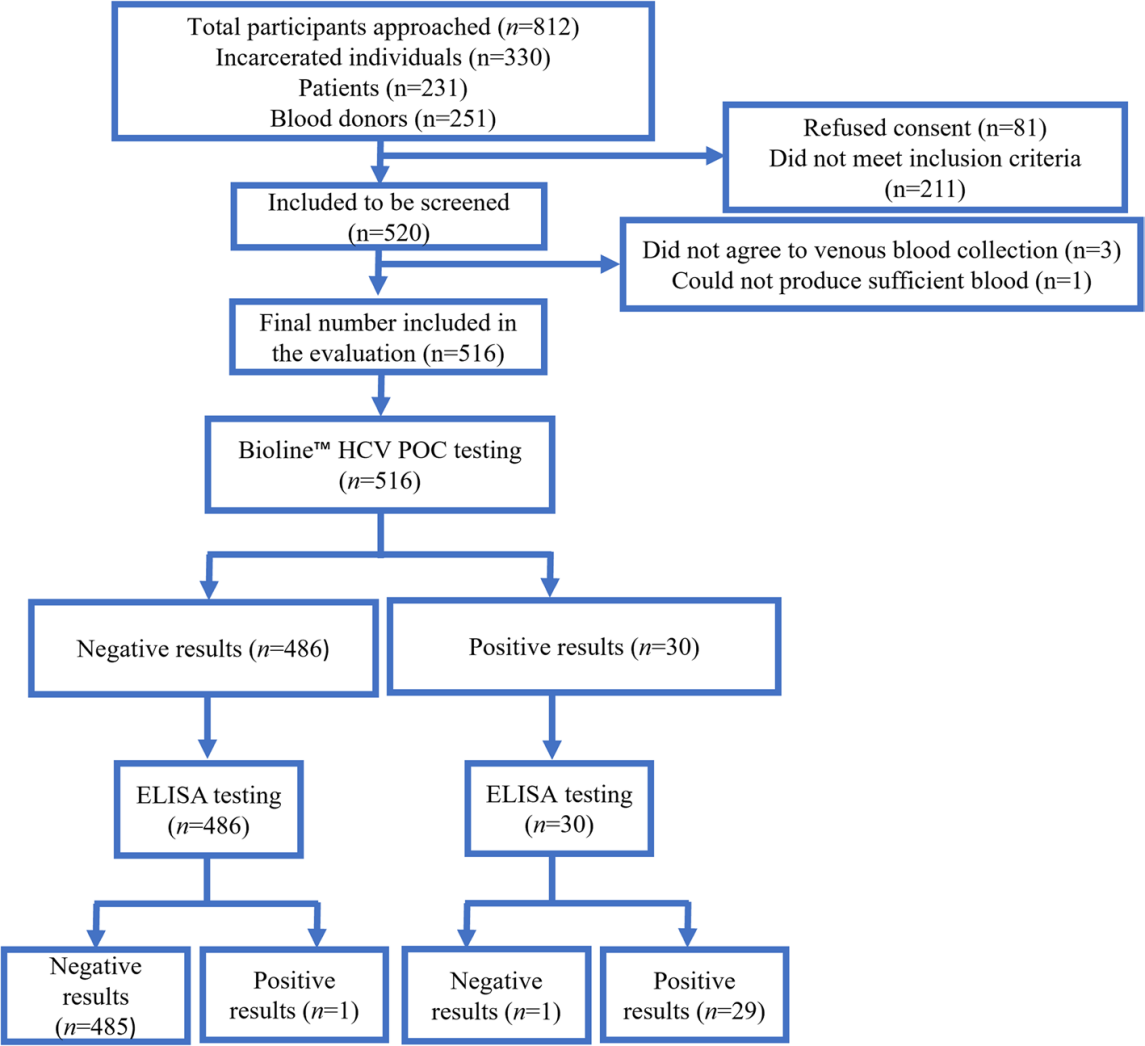
Standards for reporting of diagnostic accuracy (STARD) flowchart for evaluating the performance of the Bioline™ HCV point-of-care test in Ghana

Out of the 812 potential study participants approached, 516 met the inclusion criteria and consented to be included in the study (Fig. 3). Bioline™ HCV POC test results of 516 blood samples were compared to the results of the ELISA reference test. Both testing algorithms identified 29 true positives and 485 true negatives, with 1 false positive (ELISA negative, Bioline™ HCV POC positive) and 1 false negative (ELISA positive, Bioline™ HCV POC negative). ELISA detected 30 positives and 486 negatives, enabling assessment of Bioline™ HCV POC test performance against ELISA (Fig. 3).

The diagnostic performance of the Bioline™ HCV POC test showed high accuracy (Table 2): sensitivity (96.7%; 95% CI: 82.8–99.9%), specificity (99.8%; 95% CI: 98.9–100%), positive predictive value (96.7%; 95% CI: 82.8%–99.9%), and negative predictive value (99.8%; 95% CI: 98–100%). Test efficiency was 99.6% (95% CI: 98.6%–99.9%), with a Youden index of 0.97 (95% CI: 0.82–0.99) and an ROC area or area under curve (AUC) of 0.98 (95% CI: 0.95–1.00). Likelihood ratios were highly favorable, with LR + at 483.5 (95% CI: 414–909.1) and LR– at 0.03 (95% CI: 0.00–0.23).

**Table 2:** Summary of diagnostic performance of the Bioline™ HCV test

Diagnostic performance indicator	ꭓ(95% CI)
Sensitivity (Se)	96.7%(82.8–99.9%)
Specificity (Sp)	99.8%(98.9–100%)
Positive Predictive Value (PPV)	96.7%(82.8–99.9%)
Negative Predictive Value (NPV)	99.8%(98–100%)
Test Efficiency (TE)	99.6%(98.6–99.9%)
Youden Index (J)	0.97(0.82–0.99)
^+^ROC area or Area under curve (AUC)	0.98(0.95–1.00)
Positive Likelihood Ratio (LR +)	483.50(414.00–909.10)
Negative Likelihood Ratio (LR-)	0.03(0.00–0.23)

ꭓ estimate, *CI* Confidence interval

^+^*ROC* Receiver Operating Characteristic

**Fig. 4:**
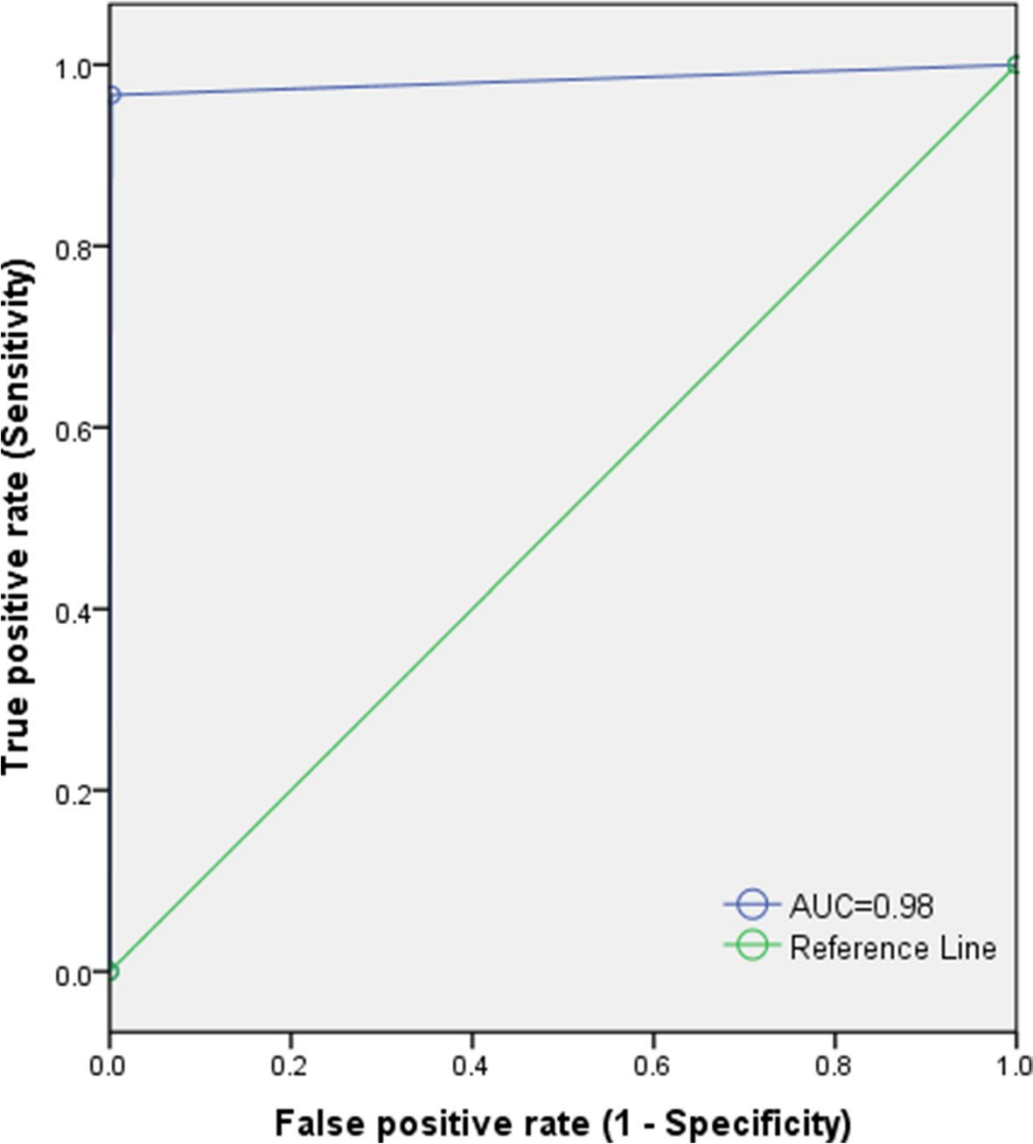
ROC curve analysis for Bioline™ HCV POC test performance

Figure 4 illustrates the diagnostic performance of the Bioline™ HCV POC test demonstrating the trade-off between the false positive and true positive rates. The ROC curve demonstrates excellent diagnostic performance, with an area under the curve (AUC) of 0.98, indicating high accuracy in distinguishing between positive and negative cases.

## Discussion

This study evaluated the performance of the Bioline™ HCV POC test in comparison to ELISA reference test, through a real-world field assessment with priority population groups in Ghana. Multiple diagnostic parameters were employed to establish population-specific performance of the test.

The results indicate that the Bioline™ HCV POC test exhibits high diagnostic accuracy, as evidenced by a sensitivity of 96.7%, specificity of 99.8%, and AUC of 0.98. These metrics highlight its reliability in identifying HCV positive and negative cases with minimal error. The high Youden index and likelihood ratios further emphasize its strong discriminatory ability, making it a valuable tool for early HCV detection. These findings are consistent with a review and meta-analysis that confirmed the effectiveness of HCV POC and rapid tests [25]. The study reported a pooled sensitivity and specificity of 98% (95% CI: 98–100%) and 100% (95% CI: 100–100%) respectively for HCV antibody rapid diagnostic tests (RDTs) when compared to an enzyme immunoassay (EIA) reference test [25]. This indicates that RDTs are highly accurate in detecting true positive and true negative cases of HCV infection, making them reliable tools for screening purposes, particularly in settings where access to laboratory-based testing is limited.

Most POC tests are designed in foreign countries, with diagnostic trials typically conducted on non-African populations before being exported to Africa. A key strength of this study is its focus on Ghanaian HCV target populations, enabling the establishment of population-specific performance metrics for the test. Furthermore, while many evaluation studies rely predominantly on sensitivity and specificity as performance indicators, this study employed a broader range of metrics (Sensitivity, Specificity, Positive Predictive Value, Negative Predictive Value, Test Efficiency, Youden Index, ROC area, Positive Likelihood Ratio (LR +), and Negative Likelihood Ratio) to provide a comprehensive assessment of the test’s performance. However, a limitation of the study is the relatively low prevalence of HCV in Ghana (1–3%), which posed challenges in obtaining sufficient positive cases for the evaluation. This challenge was addressed through active case finding within the target HCV population, resulting in an observed prevalence of 5.8%, which enhanced the robustness of the evaluation. This, therefore, had no negative impact on the performance indicators used in the evaluation. In addition, we do not seek to generalize the study findings. However, the findings are comparable and applicable to LMIC settings, particularly countries in SSA with similar geographical characteristics and HCV prevalence.

Given the high diagnostic performance of this test, future studies should explore its feasibility as a diagnostic tool rather than solely a screening test in resource-limited and remote settings. These regions often face significant challenges, including limited access to conventional confirmatory laboratories and financial barriers to confirmatory testing. Investigating this approach could offer an alternative pathway for delivering HCV diagnostic services to underserved and economically disadvantaged communities, thereby contributing to efforts aimed at promoting diagnostic equity and achieving universal health coverage.

## Conclusion

The Bioline™ HCV POC test demonstrated high diagnostic performance in this cross-sectional field evaluation, showcasing its reliability in accurately identifying true HCV positive and negative cases. This high level of diagnostic accuracy is critical for effective HCV screening and early detection, particularly in resource-limited settings where access to sophisticated laboratory infrastructure is often unavailable. Furthermore, its integration into existing healthcare systems has the potential to enhance the efficiency of HCV screening programs, contributing to the global efforts to reduce the burden of HCV. These findings highlight the Bioline™ HCV POC test as a significant advancement in overcoming diagnostic challenges, with the potential to improve health outcomes in populations most affected by HCV.

## Supplementary Information

Supplementary Material 1.

Supplementary Material 2.

Supplementary Material 3.

Supplementary Material 4.

## Data Availability

All study materials have been uploaded as supplementary information. All data generated in the study will be made available on request through the corresponding author.

## Abbreviations

HCV: Hepatitis C Virus
WHO: World Health Organization
LMICs: Low-and-middle-income countries
SSA: Sub-Saharan Africa
MSM: Men who have sex with men
PLHIV: People living with HIV
PWID: Persons who inject drugs
EIA: Enzyme Immunoassay
NAT: Nucleic acid testing
POC: Point-of-care
IVDs: Invitro diagnostics
PQ: Prequalification
PPS: Probability proportional to size
ELISA: Enzyme-linked Immunosorbent Assay
PCR: Polymerase chain reaction
Se: Sensitivity
Sp: Specificity
PPV: Positive Predictive Value
NPV: Negative Predictive Value
TE: Test Efficiency
J: Youden Index
ROC: Receiver Operating Characteristic
LR +: Positive Likelihood Ratio
LR-: Negative Likelihood Ratio
CI: Confidence interval
AUC: Area under the curve
RIBA: Recombinant immunoblot assay
