# Supplementary material for: Evaluating performance of the Bioline™ HCV point-of-care test in Ghana
Source: BMC Infect Dis. 2025 Oct 15;25:1327. doi: 10.1186/s12879-025-11730-8 (PMC12522611; doi:10.1186/s12879-025-11730-8)
Supplement: Supplementary file 1 — Supplementary Material 1. [file 12879_2025_11730_MOESM1_ESM.docx]

***The sample size for estimating the test sensitivity***

$n_{\mathrm{Se}}\geq\frac{z_{\frac{\alpha}{2}}^{2}Sⅇ\left( 1-Sⅇ \right)}{d^{2}x Prev}$

$n_{\mathrm{Se}}$ = Minimum sample size for estimating sensitivity

$Z$ = Standard normal deviate for a given level of significance (α), here α=5%, Z = 1.96

$Sⅇ$ = Pre-determined sensitivity of Bioline™ HCV= 99.3%

d= level of precision or margin of error for the sensitivity=5%

Prev= projected HCV prevalence in Ghana based on previous data= 3%

$n_{\mathrm{Se}}\geq\frac{{1.96}^{2}x 0.993\left( 1-0.993 \right)}{{0.05}^{2}x 0.03}$

$n_{\mathrm{Se}}\geq$359

***Assuming a 10% non-response rate***

$n_{\mathrm{Se}}\geq\frac{359}{1-0.1}$

$n_{\mathrm{Se}}\geq399$

***The sample size for estimating the test specificity***

$n_{\mathrm{Sp}}=\frac{z_{\frac{\alpha}{2}}^{2}\mathrm{Sp}\left( 1-Sp \right)}{d^{2}x (1-Prev)}$

$n_{\mathrm{Sp}}$ = Minimum sample size for estimating specificity

$Z$ = Standard normal deviate for a given level of significance (α), here α=5%, Z = 1.96

$\mathrm{Sp}$ = Pre-determined specificity of Bioline™ ™ HCV= 98.1%

d= level of precision or marginal of error for the specificity=5%

Prev= projected HCV prevalence in Ghana based on previous data= 3%

$n_{\mathrm{Sp}}\geq\frac{{1.96}^{2}x 0.981\left( 1-0.981 \right)}{{0.05}^{2}x 0.97}$

$n_{\mathrm{Sp}}\geq$30

***At a 10% non-response rate***

$n_{\mathrm{Sp}}\geq\frac{30}{1-0.1}$

$n_{\mathrm{Sp}}\geq34$
