# Supplementary material for: Evaluating performance of the Bioline™ HCV point-of-care test in Ghana
Source: BMC Infect Dis. 2025 Oct 15;25:1327. doi: 10.1186/s12879-025-11730-8 (PMC12522611; doi:10.1186/s12879-025-11730-8)
Supplement: Supplementary file 2 — Supplementary Material 2. [file 12879_2025_11730_MOESM2_ESM.docx]

**Supplementary file 1**

**Sampling frame and estimated sample size.**

| Stratum code | Stratum name | Population (*N_i_*) | Minimum required Sample size (*n_i_*) | Actual sample size used in the analysis |
| --- | --- | --- | --- | --- |
| HCVRG1 | Ankarful Prison Annex | 745 | 185 | 219 |
| HCVRG2 | Patients with a clinician’s request for an HCV test | 500 | 124 | 139 |
| HCVRG3 | Voluntary blood donors scheduled for pre-donation screening | 500 | 124 | 158 |
| Total | | N=1745 | *n*=433 | n=516 |
