# Supplementary material for: Evaluating performance of the Bioline™ HCV point-of-care test in Ghana
Source: BMC Infect Dis. 2025 Oct 15;25:1327. doi: 10.1186/s12879-025-11730-8 (PMC12522611; doi:10.1186/s12879-025-11730-8)
Supplement: Supplementary file 3 — Supplementary Material 3. [file 12879_2025_11730_MOESM3_ESM.docx]

**Questionnaire for data collection at the Ankarful Prison**

| **Unique identifier** |  |
| --- | --- |
| **Ward/ Block** |  |
| **Cell Number** |  |
| **Date** |  |

| 1. **Socio-demographic information** |
| --- |
| Age (years): |
| Sex: Male [ ] Female [ ] |
| Employment status before incarceration: Unemployed [ ] Employed [ ] |
| Occupation before incarceration: |
| Education status: No formal education [ ] Basic [ ] Secondary [ ] Tertiary [ ] |
| Year of incarceration: |

| 1. **Behavioural characteristics** |
| --- |
| Sexual preference/ orientation: Men [ ] Women [ ] |
| History of drug use: Yes [ ] No [ ] |
| Drug snorting: Yes [ ] No [ ] |
| Drug injection: Yes [ ] No [ ] |
| Tattoo: Yes [ ] No [ ] |
| Piercing: Yes [ ] No [ ] |
| Sharing toiletries: Yes [ ] No [ ] |

| 1. **Medical history** |
| --- |
| Known history of HCV infection: Yes [ ] No [ ] NA [ ] |
| Known history of HCV infection in a sexual partner: Yes [ ] No [ ] NA: [ ] |

| 1. **POC Test Results** |
| --- |
| HCV: |

**Thank you for taking the time to respond to this questionnaire.**
