# Supplementary material for: Evaluating performance of the Bioline™ HCV point-of-care test in Ghana
Source: BMC Infect Dis. 2025 Oct 15;25:1327. doi: 10.1186/s12879-025-11730-8 (PMC12522611; doi:10.1186/s12879-025-11730-8)
Supplement: Supplementary file 4 — Supplementary Material 4. [file 12879_2025_11730_MOESM4_ESM.docx]

**Supplementary file 3**

**Data collection tool for the Cape Coast Teaching Hospital Laboratory**

| **Date** |  | | | | |
| --- | --- | --- | --- | --- | --- |
|  | | | | | |
| **Unique identifiers** | **Age** | **Sex** | **Priosn/Patient/Donor** | **Bioline™ HCV POC results** | **ELISA results** |
|  |  |  |  |  |  |
|  |  |  |  |  |  |
|  |  |  |  |  |  |
|  |  |  |  |  |  |
|  |  |  |  |  |  |
|  |  |  |  |  |  |
|  |  |  |  |  |  |
|  |  |  |  |  |  |
|  |  |  |  |  |  |
|  |  |  |  |  |  |
